# Association of Human Cholesteryl Ester Transfer Protein-TaqI Polymorphisms with Serum HDL Cholesterol Levels in a Normolipemic Japanese Rural Population

**DOI:** 10.2188/jea.12.77

**Published:** 2007-11-30

**Authors:** Jianguo Chen, Tetsuji Yokoyama, Kyoko Saito, Nobuo Yoshiike, Chigusa Date, Heizo Tanaka

**Affiliations:** 1 Department of Epidemiology, Medical Research Institute, Tokyo Medical and Dental University, 2-3-10 Kanda-Surugadai, Chiyoda-ku, Tokyo 101-0062, Japan.; 2 Division of Adult Health Science, National Institute of Health and Nutrition, Tokyo, Japan.; 3 Department of Public Health, Osaka City University School of Medicine, Osaka, Japan.

**Keywords:** cholesteryl ester transfer protein, HDL, polymorphism, Japanese population

## Abstract

We examined allele frequencies for the common cholesteryl ester transfer protein (CETP) TaqI polymorphisms and the associations of CETP-TaqI polymorphisms with serum lipid and lipoprotein levels taking into account for selected lifestyle factors in a well-characterized random sample of 527 healthy subjects living a rural community in Japan (256 men and 271 women aged 40-69 years). B2 allele frequency was 0.39 in men and 0.41 in women, and its presence was significantly associated with increased levels of HDL cholesterol (HDL-C) in men (P=0.003 for linear trend). A similar tendency in women was observed, although P value for trend did not reach 0.05. There were not significant interactions between TaqIB genotype and smoking and alcohol drinking or daily physical activity in HDL-C. There were no statistically significant differences among TaqIA genotype in lipid and lipoprotein levels. Multiple linear regression analysis showed that B1B2 and B2B2 explained 1.7% and 2.2%, and 0.6% and 1.0% of variation in men and in women in HDL-C, respectively. We conclude that the CETP-TaqIB polymorphism has a quantitative influence, but appears to be stronger one in men, on HDL-C levels even after adjustment for important lifestyle factors (smoking, alcohol drinking, and daily physical activity).

One of the potential antiatherogenic mechanisms of HDL cholesterol (HDL-C) is considered that HDL-C plays a function in the “reverse cholesterol transport” (RCT) from extrahepatic tissues to the liver^1^^-^^3^^)^. The RCT involves the transfer of cholesteryl esters from HDL to VLDL and LDL by a cholesteryl ester transfer protein (CETP) in exchange for triglycerides^2^^,^^4^^,^^5^^)^. Human CETP is a plasma glycoprotein of Mr74 000, and is expressed primarily in liver, spleen, adipose tissue, small intestine, adrenal gland, kidney, skeletal muscle, and heart^5^^,^^6^^)^. The CETP gene spans ≈ 25 000 base pairs, is composed of 16 exons interrupted by 15 introns of variable size, and has been localized on chromosome 16q21 adjacent to the lecithin-cholesterol acyltransferase (LCAT) gene^6^^)^.

Several mutations that cause CETP deficiency are associated with marked hyperalphalipoproteinemia that has been associated with an increase risk of coronary heart disease (CHD) in Japanese subjects^7^^,^^8^^)^. However, the effects of more common restriction fragment length polymorphisms (RFLPs) in the CETP gene to CHD risk factor levels and atherosclerosis are unclear in Japanese population. Conversely, several RFLPs have been reported very well in the West. The most studied DNA polymorphisms of CETP gene have been TaqIB^9^^-^^24^^)^, which affects 277^th^ nucleotide in the first intron of the gene. In many of these studies, individuals carrying the B2 allele (absence of the TaqI restriction site) at this polymorphic site had the highest HDL-C concentrations^10^^-^^17^^,^^19^^,^^21^^-^^24^^)^ in normolipemic white subjects. But, studies in Finland^18^^)^, Italian migrants to Australia^19^^)^, and healthy French Canadians^20^^)^ found no association between the CETP-TaqIB polymorphism and HDL-C levels. It has been suggested that the association of CETP-TaqIB polymorphism with HDL-C levels may be complicated by environmental factors, such as smoking^10^^,^^12^^,^^25^^)^ and alcohol drinking^13^^,^^14^^)^.

There are two RFLPs at CETP locus referred to as polymorphism A (intron 10) and B by using the restriction enzyme TaqI. Two studies found that the TaqIA RFLP was also associated with HDL-C levels in Caucasian females^19^^,^^20^^)^.

In the present cross-sectional study, we have selected the TaqI RFLPs in the CETP gene to estimate allele frequencies of the TaqI polymorphisms and to determine the association of this DNA marker with serum lipid and lipoprotein levels in a normolipemic Japanese rural population. The influences of environmental risk factors, such as smoking, alcohol drinking, individual physical activity on the extent of this association are also taken into consideration.

## METHODS

### Subjects

The present study was part of work being carried out to continuously monitor the changes in lifestyles and risk factors of cardiovascular diseases during the last decade in Shiso, a rural county located in the northwestern part of Hyogo Prefecture, Japan^26^^)^. This county consisted of Y, I, S, H, and C towns. The total populations of Shiso through all age were 25912 in men and 27933 in women, and those in the age of 40-69 years were 10485 (5238, 2189, 1185, 1044, and 829 in Y, I, S, H, and C towns, respectively) in men and 10615 (5343, 2253, 1157, 1031, and 831, respectively) in women, at time of our survey in 1999 (Y and H towns) or in 2000 (I, S, and C towns). Considering all residents aged 40-69 years as the eligible population, the subjects were selected by stratified random sampling based on town, sex, and decade of age so that the expected number of participants in each stratum would be 35 (Y and I towns) or 20 (H, S, and C towns) and hence 390 men and 390 women in total aside from the non-responders. Because the response rate was estimated to be 75% based on previous studies in this area, we recruited 520 men and 520 women in total (overall sampling rate: 5.0% and 4.9%, respectively). Of these, 358 men and 393 women actually participated in this study (overall response rate: 68.8% and 75.6%, respectively). There was no marked difference in the response rate among the 5 towns.

All participants were examined at local community halls. Under supervision of dietitians or nurses specifically trained for this study, each subject donated blood sample into 10 ml EDTA tubes after a 12-hour fast and completed standardized questionnaires, which asked for smoking and alcohol consumption habits, degree of physical activity, medical history (hypertension, hyperlipidemia, diabetes, cardiovascular disease), and medical use for lipid-lowering or antihypertensive drugs. We excluded those who were taking lipid-lowering drugs (32 men and 41 women) or were non-fasting (65 men and 75 women), respectively. We could not get genomic DNA from blood cells from 8 subjects (3 men and 5 women). As a result, 530 subjects (258 men and 272 women) were included in this study. Of these, TaqIB and TaqIA genotypes were not determined in 3 and 4 subjects, respectively. Height and weight were measured, and body mass index (BMI) was defined as weight (kg)/height^2^ (m).

Ethical approval for this study was granted by the ethics review committee of Medical Research Institute, Tokyo Medical and Dental University. All participants provided written informed consent.

### Laboratory Methods

Venus blood samples were immediately transported at 4°C to our lab. Serum was obtained by centrifugation at 3000 rpm for 10 min at 4°C for measurement of lipids, and blood cells were frozen at -80°C until analysis. Total cholesterol (TC), HDL-C, and triglyceride (TG) concentrations were determined by an enzymatic method on an autoanalyzer (Hitachi 7170), and LDL cholesterol was calculated by Friedewald’s method^27^^)^. Standardization of lipid measurements was achieved in the light of the Lipid Standardization Program of the US Centers for Disease Control and Prevention through the Osaka Medical Center for Cancer and Cardiovascular Disease, Japan^28^^)^.

Genomic DNA was prepared from separated leukocytes from frozen blood cells by using Puregene (Gentra Systems, Inc). Determinations of the TaqI polymorphisms with polymerase chain reaction were originally described by Draya and Kuivenhoven^9^^,^^15^^)^.

### Assessment of life-style factors

Smokers were individuals who were currently smoking, and were grouped: 1) smokers <20 cigarettes/day and 2) smokers≧20 cigarettes/day. Nonsmokers were those who had never smoked or were ex-smokers but had stopped for more than 1 year. Alcohol consumption included the principal forms in Japanese, which were beer, wine, whisky, sake and shouchu. The drinking status was classified by frequency of alcohol drinking regardless of the sorts of drinking as follows: 1) drinkers who had an alcohol drinking habits and were currently drinking for more than once a week and 2) nondrinkers who had never drunk or had not drunk for more than two weeks.

We designed a standardized questionnaire to assess individual degree of physical activity both for leisure-time and on-the-job-time during the latest one year. The duration and frequency of each activity were asked. The categories of intensity of each activity were set according to the methods of a NHLBI workshop^29^^)^ and expressed as METs. In this study we used the ‘Active Intensity Index’ (AII) that was average METs during the time except sleeping hours for assessment of individual degree of physical activity in daily life.

### Statistical analysis

Subjects were divided into groups according to the genotypes, status of smoking or alcohol drinking, and tertiles of AII for each sex. Genotypic data were coded as 0, 1, and 2 according to the number of the less frequent allele for most analyses. This ordinal 0, 1, 2 variable was employed to test an allele-dose linear effect, which is equivalent to assuming codominance of the genetic effect^14^^)^. Owing to very few smokers in women, analysis of smoking was done only in men. Smoking and alcohol drinking were coded as 0 (nonsmokers or nondrinkers) and 1 (smokers or drinkers). Tertiles of AII were coded as 1, 2, and 3.

We used χ^2^ tests for categorical measurements and 2-sample t tests for continuous measurements to compare men and women. The allele frequency was determined by direct counting, which provides maximum-likelihood allele-frequency estimates for a locus with all codominant alleles^30^^)^. χ^2^ analysis with 1 *df* was used to test for any deviation of the CETP gene variants from the Hardy-Weinberg equilibrium^31^^)^. The degree of linkage disequilibrium, D, between TaqIA and TaqIB was estimated by maximum likelihood from the frequency of diploid genotypes, and was tested by χ^2^ analysis proposed by Hill^32^^)^. Data were tested for normality using normal probability plots, and if necessary, transformed to produce a normal distribution. BMI and TG were log transformed to reduce the skewness in distribution, but the non-transformed values are presented in the tables. The ratio of TC to HDL-C was used as a dependent variable. Associations of lipid and lipoprotein levels with the genotypes and lifestyle variables were estimated by analysis of variance (ANOVA) using the general linear models (GLM) procedure with F tests computed from the Type III sums of squares^33^^)^, controlling for age, log BMI, smoking, alcohol drinking, AII, and fasting triglyceride (because of its strong association with HDL-C^34^^)^). The Least-Square Means from GLM procedure were used to determine the level of significance in pairwise comparisons between genotype classes. The Tukey post hoc test was done in conditions where a significant group effect was found. P values for linear trends across categories were calculated by ANOVA^33^^)^. Two-way interactions between genotype and smoking, alcohol drinking, and AII in determination of HDL-C levels were also tested using GLM procedure for unbalance ANOVA^33^^)^.

The independent contributions and the extents of the association of the TaqIB genotype (dummy variables), age (years), log BMI, smoking (smokers, nonsmokers), alcohol drinking (drinkers, nondrinkers), AII (tertiles of AII used as dummy variables), and log fasting triglyceride to HDL-C levels were assessed using the multiple linear regression analysis. Partial R^2^ value for a given independent variable was used to estimate the percentage of variance in HDL-C levels explained by that independent variable^33^^)^. All analyses were performed by the SAS statistical software package (release 6.12, SAS Institute). A two-sided P value <0.05 was considered significant.

## RESULTS

### Subject characteristics

Table 1 provides a summary of the demographic and biochemical characteristics of the study subjects according to sex. The mean age and active intensity index are similar between men and women. The mean TC, HDL-C, and LDL-C levels are significantly higher in women than in men. However, the mean log TG levels and TC/HDL-C ratio are significantly higher in men than in women. Men are more overweight than women are. More than 80% of men and 30% of women drink regularly (>once/week). Proportion of smokers is very low in women (only 11 persons), and is over 50% in men.

**Table 1.** Demographic and biochemical characteristics of the study subjects according to sex.

Variable	Men (n=256)		Women (n=271)		P^a^
Age, years	54.8	(8.6)	54.0	(8.6)	0.241
BMI, kg/m^2^	23.3	(3.1)	22.6	(2.8)	0.004
TC, mg/dl	198.8	(32.8)	210.4	(34.3)	<0.001
HDL-C, mg/dl	55.8	(14.1)	63.3	(14.2)	<0.001
LDL-C, mg/dl	118.0	(31.4)	127.9	(32.0)	<0.001
TG, mg/dl	126.6	(75.8)	96.6	(54.9)	<0.001
TC/HDL ratio	3.8	(1.0)	3.5	(0.9)	0.001
Drinker, %	81.3		31.7		<0.001
Smoker, %	54.7		4.1		<0.001
Active intensity index, METs	2.0	(0.4)	2.0	(0.3)	0.361

Values are means (SD) or percentages.

^a^ Men vs women.

### Genotypes and Frequencies

The prevalences of TaqI polymorphisms and allele frequencies are shown in Table 2. There are no significant differences in the frequencies of the A2 and B2 alleles between men and women, and the distributions of alleles are consistent with Hardy-Weinberg equilibrium. We find a significant association between the two polymorphisms (D=0.02, χ^2^ =13.5, P<0.001). This finding is in agreement with previous reported results^10^^,^^15^^,^^25^.

**Table 2.** Prevalence of TaqI polymorphisms at the CETP locus.

Genotype	All			Men			Women		
		
	Obs (%)		Exp	Obs (%)		Exp	Obs (%)		Exp
TaqIA	526			255			271		
A1A1	455	(86.5)	454.6	219	(85.9)	218.3	236	(87.1)	236.3
A1A2	68	(12.9)	68.8	34	(13.3)	35.4	34	(12.6)	33.4
A2A2	3	(0.6)	2.6	2	(0.8)	1.4	1	(0.4)	1.2
A2 allele frequency	0.07			0.08			0.07		
χ^2^	0.07			0.31			0.04		
P	>0.70			>0.50			>0.70		

TaqIB	527			256			271		
B1B1	180	(34.2)	189.7	89	(34.8)	95.3	91	(33.6)	94.3
B1B2	272	(51.6)	253.0	134	(52.3)	121.8	138	(50.9)	131.1
B2B2	75	(14.2)	84.3	33	(12.9)	38.9	42	(15.5)	45.6
B2 allele frequency	0.40			0.39			0.41		
χ^2^	2.85			2.53			0.76		
P	>0.05			>0.05			>0.30		

Obs: observed number of genotypes; Exp: expected number of genotypes by Hardy-Weinberg equilibrium.

χ^2^ test for any deviation from the Hardy-Weinberg equilibrium.

### Association of TaqIB polymorphism with lipid and lipoprotein levels

Table 3 shows that in men or women, there is no significant association of TaqIA genotype with levels of TC, HDL-C, LDL-C, TG, and TC/HDL-C ratio. However in men, the B2 allele is significantly associated with increased levels of HDL-C. B2B2 homozygotes are significantly associated with a 13.7% (P<0.01) increase in HDL-C levels compared with B1B1 homozygotes. B1B2 heterozygotes have intermediate levels. In women, there is an increased trend in HDL-C from B1B1 to B2B2, although the P value for linear trend does not reach statistically significant level (0.05). There are not associations between CETP-TaqIB genotype and increased levels of LDL-C, TC/HDL-C ratio, and TG both in men and in women. An unexpected significant association between TaqIB genotype and increased TC levels appears probably because of a positive correlation between HDL-C and TC (Spearman correlation coefficient=0.10 P<0.03, data not shown).

**Table 3.** Serum levels of lipids, lipoproteins of the subjects according to CETP-TaqI genotypes.

Variable	CETP-TaqIB Genotype							CETP-TaqIA Genotype				
	
	B1B1		B1B2		B2B2		Test for linear trend^a^	A1A1		A1A2		Test for linear trend^a^
Men												
n	89		134		33			219		34		
TC, mg/dl	192.8	(3.3)	199.2	(2.7)	213.7	(5.4)*†	0.005	197.6	(2.1)	204.7	(5.5)	0.242
HDL-C, mg/dl	52.6	(1.2)	56.9	(1.0) ‡	59.8	(2.0)*	0.003	55.7	(0.8)	56.5	(2.1)	0.693
LDL-C, mg/dl	115.2	(3.2)	117.1	(2.6)	128.7	(5.3)	0.088	116.9	(2.0)	122.8	(5.3)	0.312
TG, mg/dl	125.2	(7.4)	129.2	(6.0)	119.6	(12.1)	0.599	128.9	(4.7)	113.7	(12.1)	0.144
TC/HDL-C ratio	3.8	(0.1)	3.7	(0.1)	3.9	(0.1)	0.237	3.7	(0.1)	3.9	(0.1)	0.364

Women												
n	91		138		42			236		34		
TC, mg/dl	207.4	(3.4)	210.0	(2.7)	219.1	(5.0)	0.145	210.6	(2.1)	212.3	(5.6)	0.826
HDL-C, mg/dl	61.4	(1.3)	63.7	(1.0)	65.7	(1.9)	0.138	63.1	(0.8)	64.0	(2.1)	0.642
LDL-C, mg/dl	126.8	(3.2)	127.0	(2.6)	133.7	(4.7)	0.382	128.1	(2.0)	128.8	(5.4)	0.968
TG, mg/dl	93.2	(5.4)	99.2	(4.4)	95.6	(7.9)	0.642	98.0	(3.3)	89.8	(8.9)	0.393
TC/HDL-C ratio	3.6	(0.1)	3.4	(0.1)	3.4	(0.1)	0.455	3.5	(0.0)	3.5	(0.1)	0.866

Values are Least-Squares Means (SE) adjusted for the age, log BMI, smoking, alcohol drinking, active intensity index, fasting triglyceride (except TG).

Shown at significant differences between B2B2 and B1B1 (*), B2B2 and B1B2 (†), and B2B1 and B1B1 (‡).

^a^ The number of alleles 2 (coded as 0, 1, 2) is used as a continuous variable and tested in a GLM with the variable in the left side column as dependent variable.

### The influences of selected lifestyle factors on the association of CETP-TaqIB genotype with HDL-C levels

Table 4 shows that in men, the significant association of the TaqI B2 restriction site with higher HDL-C levels is seen only in the nonsmokers (P=0.043), alcohol drinkers (P=0.017), and those with the lowest tertile of AII (P=0.020). But there is a similar increased tendency in HDL-C from B1B1 to B2B2 also in smokers (P=0.052), nondrinkers (P=0.075), and the subjects with the highest tertile of AII (P=0.071) in men. In women, there is an increased trend contingent upon addition of B2 allele in alcohol drinkers and tertile 3 of AII group, although P value for trend does not reach 0.05. Within each genotype, nonsmokers have higher HDL-C levels than smokers do. In both sexes, alcohol drinkers have higher HDL-C levels than nondrinkers do.

**Table 4.** Serum high-density lipoprotein cholesterol levels according to CETP-TaqIB genotype by status of smoking (only men) and alcohol drinking and tertiles of active intensity index (AII).

	Nonsmokers		Smokers		Nondrinkers		Drinkers		Tertile 1 of AII		Tertile 2 of AII		Tertile 3 of AII	
						
	n	LSM (SE)	n	LSM (SE)	n	LSM (SE)	n	LSM (SE)	n	LSM (SE)	n	LSM (SE)	n	LSM (SE)
HDL-C, mg/dl														
Men														
B1B1	43	53.4 (2.0)	46	51.7 (1.5)	17	44.4 (2.4)	72	54.6 (1.4)	34	49.5 (1.9)	21	57.1 (2.4)	34	53.5 (2.2)
B1B2	59	59.4 (1.7)‡	75	55.0 (1.2)	29	50.4 (1.8)	105	58.1 (1.2)	50	56.5 (1.6)‡	43	57.4 (1.7)	41	57.0 (2.0)
B2B2	14	61.7 (3.6)*	19	58.2 (2.4)*	2	57.5 (7.7)	31	61.7 (2.2)*	29	55.3 (2.9)	5	58.5 (5.2)	13	63.2 (3.5)
P for trend^a^		0.043		0.052		0.075		0.017		0.020		0.973		0.071
P^b^				0.914				0.503				0.454		

women														
B1B1					68	60.8 (1.4)	23	62.3 (2.8)	32	59.4 (2.3)	33	61.9 (2.2)	26	62.5 (2.4)
B1B2					90	62.0 (1.2)	48	67.4 (1.9)	37	61.5 (2.1)	56	65.1 (1.7)	45	64.5 (1.8)
B2B2					27	62.6 (2.2)	15	71.5 (3.5)	14	63.8 (3.5)	16	63.3 (3.1)	12	70.6 (3.5)
P for trend^a^						0.743		0.154		0.655		0.511		0.181
P^b^								0.222				0.399		

Values are Least-Square Means (SE) adjusted for the age, log BMI, smoking (except status of smoking), alcohol drinking (expect status of alcohol drinking), AII (expect tertiles of active intensive index), fasting triglyceride (expect TG).

Tertile 1=1.50 to <1.83 METs; Tertile 2=1.83 to <2.07 METs; Tertile 3=2.07 to 3.84 METs.

Shown at significant differences between B2B2 and B1B1 (*), B2B2 and B1B2 (†), and B2B1 and B1B1 (‡).

^a^ The number of alleles 2 (coded as 0, 1, 2) and tertiles of AII (coded as 1, 2, 3) are used as continuous independent variables. Smoking or alcohol drinking status is coded as 0, 1.

^b^ Test for the interactions between CETP-TaqIB genotype and each of smoking, alcohol drinking, and AII.

Each of selected lifestyle factors (smoking, alcohol drinking, and AII) is assessed for its interaction with TaqIB genotype for HDL-C levels (Table 4). We do not find any significant interaction both in men and in women.

### Multiple regression analysis

In men, B1B2 heterozygotes and B2B2 homozygotes have independent effects on HDL-C (Table 5). The regression coefficients for B1B2 and B2B2 are 4.1 (95% CI 1.0 to 7.2) mg/dl and 6.9 (95% CI 2.4 to 11.4) mg/dl accounting for 1.7% and 2.2% of the variability in HDL-C, respectively. Log BMI, smoking, alcohol drinking, and log fasting TG are also independently associated with HDL-C. The final model explains 37.4% (P<0.01) of the variation in HDL-C. In women, log BMI, alcohol drinking, and log fasting TG are significant contributors. B2B2 is marginally significant dedicator (P=0.06). B1B2 explains 0.6% of variation in HDL-C.

**Table 5.** Results of multiple linear regression analysis showing the relative contributions and extents of the relation of CETP-TaqIB genotype, selected lifestyle factors, and relative parameters to serum HDL-C levels.

Dependent Variable	Independent Variable	Men			Women		
	
		β	PR^2^	P	β	PR^2^	P
HDL-C, mg/dl	Age (years)	-0.1 (0.1)	0.5	0.14	0.0 (0.1)	0.0	0.75
	Log BMI	-20.5 (6.0)	3.0	<0.01	-25.0 (6.3)	4.1	<0.01
	CETP-TaqIB Genotype						
	B1B1	reference			reference		
	B1B2	4.1 (1.6)	1.7	<0.01	2.3 (1.7)	0.6	0.15
	B2B2	6.9 (2.3)	2.2	<0.01	4.3 (2.3)	1.0	0.06
	Smoking						
	Nonsmokers	reference			reference		
	Smokers	-4.1 (1.5)	2.0	<0.01	2.8 (3.8)	0.1	0.45
	Alcohol Drinking						
	Nondrinkers	reference			reference		
	Drinkers	9.6 (1.9)	6.9	<0.01	4.4 (1.6)	2.0	<0.01
	Tertiles of AII (METs)						
	Tertile 1	reference			reference		
	Tertile 2	1.6 (1.8)	0.2	0.39	1.6 (1.8)	0.2	0.37
	Tertile 3	-0.1 (1.7)	0.0	0.93	1.8 (1.9)	0.2	0.36
	Log TG	-11.3 (1.5)	14.0	<0.01	-12.0 (1.7)	13.4	<0.01

β: regression coefficient (SE); PR^2^: partial R^2^ expressed as percentages; TG: fasting triglyceride; AII: active intensity index (METs). CETP-TaqIB genotype and tertiles of AII are used as dummy variables to evaluate the effects of B1B2, B2B2, tertile 2, and tertile 3 to HDL-C, respectively.

Status of smoking or alcohol drinking is coded as 0, 1.

## DISCUSSION

In this cross-sectional study, using an enough large well-characterized random sample, which had homogeneous ethnic background and place of residence, we first described the associations between CETP-TaqI polymorphisms and lipid and lipoprotein levels in a normolipemic Japanese rural population. Data suggested that B2B2 genotype of the TaqIB polymorphism in the CETP gene be independently associated with HDL-C concentrations in assuming codominance of the genetic effect even after adjustment for fasting TG and other environmental factors, particularly in men. Screening of all study subjects for the TaqIB polymorphism revealed that the frequencies were similar to white subjects, indicating that this marker is a common genetic variation also among Japanese population. No statistically significant associations in lipid and lipoprotein levels were observed among TaqIA genotype.

The mechanism for influence of TaqIB-CETP polymorphism on HDL-C levels is not very clear. This polymorphism affects the 277^th^ nucleotide in intron 1 and should not affect the splicing of the CETP mRNA, so the CETP activity is not directly affected by this polymorphism^12^^-^^14^^,^^36^^)^. In previous studies^13^^-^^15^^)^, the B2 allele was significantly associated with lower CETP levels and higher HDL-C levels, but HDL-C levels were not significantly correlated with CETP levels. For this reason, Fumeron et al suggested that the influence of CETP-TaqIB polymoiphism on HDL-C levels be independent of its effect to CETP levels^14^^)^. In the present study, we had not measurement on CETP levels/activities. A recent study in ECTIM (using healthy control only) demonstrated that -629A/C-CETP polymorphism, which was in the promoter region of the CETP gene, was functional variant, almost complete linkage disequilibrium with the TaqIB polymorphism, and significantly associated with HDL-C levels^36^^)^. Although LACT gene is also located on chromosome 16 adjacent to the CETP gene^6^^)^, no associations have been found between LCAT activity and the CETP-TaqIB polymorphism and HDL-C levels^11^^)^.

HDL-C is positively correlated with TC, and the degree of this correlation is consistent with the proportion of the TC carried in the HDL fraction^37^^)^. In this population, there was also a positive correlation between HDL-C and TC levels (Spearman correlation coefficient=0.10, P<0.03). Therefore, the unexpected association of CETP-TaqIB genotype with TC found in this study maybe just reflected the results of the correlation between TC and HDL-C.

Many epidemiological evidences have demonstrated that environmental factors such as smoking, alcohol drinking, and physical activity are clearly involved in the regulation of HDL-C levels^38^^,^^39^^)^. For this reason, in this study we also investigated the possible influences of selected environmental factors on the genetic regulation of HDL-C levels. There was a significant association between CETP-TaqIB polymorphism and HDL-C levels in male nonsmokers, alcohol drinkers, and those doing relatively light physical activity. But, a similar association was observed also in smokers, nondrinkers, and the highest tertile of AII group, although P value did not attain statistically significant level. We did not find any statistically significant interaction terms between TaqIB genotype and environmental factors (smoking, alcohol drinking, and daily physical activity). Therefore, we did not positively confirm that the association of CETP-TaqIB polymorphism with HDL-C could be modified by lifestyle factors. Several studies found this association could be influenced by environmental factors, such as smoking^10^^,^^12^^,^^19^^)^ and alcohol drinking^14^^)^. This inconsistency may be due to variations of covariates such as sex, BMI, sample size, study design, and other environmental factors.

Kauma et al.^21^^)^ found that the significant association between CETP-TaqIB genotype and increased HDL-C appeared only in women in a Finnish population. However, we were unable to determine the sex interaction in this Japanese population. We hypothesized that sex hormones may play a role in the observed sex differences besides different lifestyle factors. Women have higher levels of CETP than men do and increased CETP levels during the late pregnancy^40^^)^. This suggests that sex hormones have an effect to CETP expression.

Studies in Finland^18^^)^, Italian migrants to Australia^19^^)^, and healthy French Canadians^20^^)^ did not find the association of CETP-TaqIB polymorphism with HDL-C. This might be due to a common feature, namely a very small sample size and unavailable information about environmental factors in these studies.

In conclusion, we have shown that in this normolipemic Japanese rural population, CETP-TaqIB polymorphism is a common genetic variant and independently associated with increased HDL-C levels. This DNA marker is a good predictor for HDL-C, but appears to be stronger one in men. The effect of this DNA marker to atherosclerotic cardiovascular disease should be distinguished in the future. Although the present study permits only imagination about the potential mechanisms, our findings indicate that environmental factors may conceal important genetic information when doing the single or multiple RFLPs association study approach in modulating levels of atherosclerotic risk traits in the general population.
